# DNA Methylation and Transcription in a Distal Region Upstream from the Bovine AlphaS1 Casein Gene after Once or Twice Daily Milking

**DOI:** 10.1371/journal.pone.0111556

**Published:** 2014-11-04

**Authors:** Minh Nguyen, Marion Boutinaud, Barbara Pétridou, Anne Gabory, Maëlle Pannetier, Sophie Chat, Stephan Bouet, Luc Jouneau, Florence Jaffrezic, Denis Laloë, Christophe Klopp, Nicolas Brun, Clémence Kress, Hélène Jammes, Madia Charlier, Eve Devinoy

**Affiliations:** 1 INRA, UMR1313 Génétique Animale et Biologie Intégrative, Jouy-en-Josas, France; 2 INRA, UMR1348 Physiologie Environnement et Génétique pour l′Animal et les Systèmes d′Elevage, Saint-Gilles, France; 3 INRA, UMR1198 Biologie du Développement et Reproduction, Jouy-en-Josas, France; 4 INRA, Sigenae, UR875 Biométrie et Intelligence Artificielle, Castanet-Tolosan, France; 5 INSERM U846 Stem Cell and Brain Research Institute, INRA, USC1361 AGROBIOSYSTEM, Université de Lyon 1 UMR S 846, Bron, France; Institute of Farm Animal Genetics, Germany

## Abstract

Once daily milking (ODM) induces a reduction in milk production when compared to twice daily milking (TDM). Unilateral ODM of one udder half and TDM of the other half, enables the study of underlying mechanisms independently of inter-individual variability (same genetic background) and of environmental factors. Our results show that in first-calf heifers three CpG, located 10 kb upstream from the *CSN1S1* gene were methylated to 33, 34 and 28%, respectively, after TDM but these levels were higher after ODM, 38, 38 and 33%, respectively. These methylation levels were much lower than those observed in the mammary gland during pregnancy (57, 59 and 50%, respectively) or in the liver (74, 78 and 61%, respectively). The methylation level of a fourth CpG (CpG4), located close by (29% during TDM) was not altered after ODM. CpG4 methylation reached 39.7% and 59.5%, during pregnancy or in the liver, respectively. CpG4 is located within a weak STAT5 binding element, arranged in tandem with a second high affinity STAT5 element. STAT5 binding is only marginally modulated by CpG4 methylation, but it may be altered by the methylation levels of the three other CpG nearby. Our results therefore shed light on mechanisms that help to explain how milk production is almost, but not fully, restored when TDM is resumed (15.1±0.2 kg/day instead of 16.2±0.2 kg/day, p<0.01). The STAT5 elements are 100 bp away from a region transcribed in the antisense orientation, in the mammary gland during lactation, but not during pregnancy or in other reproductive organs (ovary or testes). We now need to clarify whether the transcription of this novel RNA is a consequence of STAT5 interacting with the CSN1S1 distal region, or whether it plays a role in the chromatin structure of this region.

## Introduction

Once daily milking (ODM) induces both a short term and long lasting reduction in milk production, when compared to twice daily milking (TDM) [1], [2]. These effects are mediated by systemic endocrine regulations but also involve some local regulation [2]. In the lactating cow, each udder half or udder quarter can be milked separately, for example once daily or twice daily. Milk production, as well as the development of mammary tissue in the quarters, can then be compared and the local effects induced by ODM analysed [2]. The main advantage of this animal model is to enable the comparison of effects induced by differential milking in mammary samples which have the same genome, are subject to the same endocrine regulations and are in the same environment.

Local and mild inflammation has been observed during ODM [3]. In turn, inflammation of the bovine mammary gland observed during involution or induced by mastitis has been shown to reduce milk production [4]–[7].

**Figure 1:**
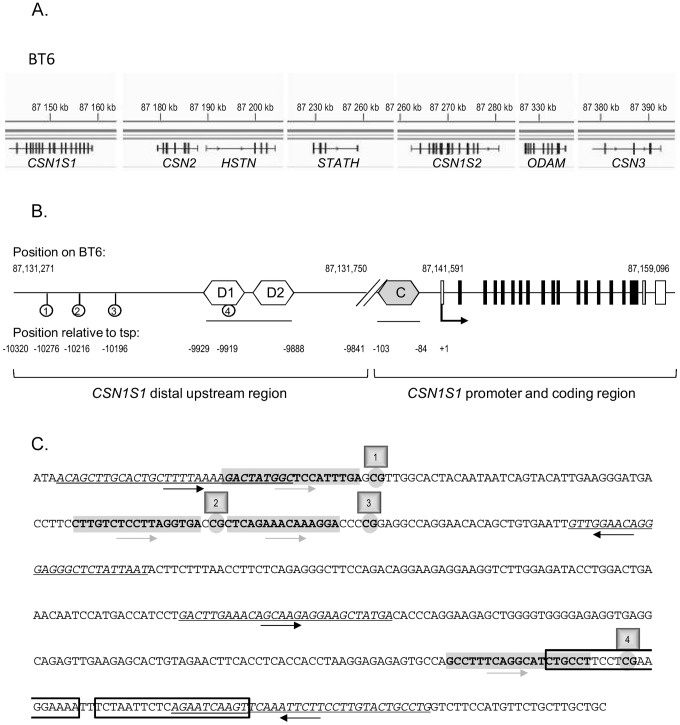
Description of the bovine *CSN* gene cluster and the distal upstream region of *CSN1S1*. **(A)** The bovine *CSN* gene cluster, located on BT6 from position 87,140 kb to 87,395 kb (Ensembl release 75, February 2014), is shown at the top of the Figure. Transcripts are indicated below. **(B)** Schematic representation of the bovine *CSN1S1* gene and its upstream region on BT6. The transcription start point (tsp, +1) of the bovine *CSN1S1* gene is located at position 87,141,591 [34]. The distal upstream regulatory region depicted in this Figure is located between 87,131,271 and 87,131,750. It extends from 10,320 to 9,841 nt upstream from the tsp. The *CSN1S1* coding exons are depicted by black boxes, and the 3′ and 5′ untranslated regions by white boxes. Two distal potential STAT5 binding sequences (D1 and D2) are indicated by white hexagons, the proximal consensus STAT5 binding site (C) is indicated by a grey hexagon. The four CpG are indicated by lollipops. **(C)** Sequence of the *CSN1S1* distal upstream region depicted in (B). Sequences which were used to design PCR primers or pyrosequencing primers are italicized and underlined or are in bold and highlighted in grey, respectively. Their orientation is indicated by arrows. The four C studied (within the CpG sequence) located at −10276, −10216, −10196 and −9919 from the tsp previously described [34] and at positions 87.131.315; 87.131.375; 87.131.395 and 87.131.672 on BT6 are indicated in bold and circled. The two potential STAT5 binding sites from −9929 to −9910 and from −9907 to −9888 are boxed.

Milk is very rich in caseins (CSN) which are the principal milk proteins. They are encoded by four to five genes depending on the species: *CSN1S1*, *CSN2*, *CSN1S2* (one or two copies) and *CSN3*. The *CSN* genes are located with a few other genes (*HSTN, STATH and ODAM)*, within a 250 kb cluster on chromosome 6 in the bovine genome (Figure 1A) [8]. *CSN1S1* is the more distal upstream gene in this cluster. These *CSN* genes, some of which are really close to each other, have been shown to be globally co-regulated in both mice and cattle [9]–[11] and to reach maximum levels of expression during lactation. Their regulatory regions, and the epigenetic marks which control gene expression at different physiological stages *in vivo* or *in vitro*, have been analysed [4], [12]–[14]. However, regions which allow the specific expression of *CSN* genes in the mammary gland, as opposed to those of *HSTN, STATH and ODAM* which are also detected in other tissues, have yet to be defined. The underlying mechanisms may also involve nuclear organization of the chromatin loops in mammary nuclei [13], and the epigenenetic state of the whole cluster [13], [14].

Among all epigenetic marks, DNA methylation is a relatively stable event which in most cases is passively erased after cell division and which has been only described as being actively erased in a few models such as breast cancer cell lines [15], [16], during early embryo development [17] or neuronal development [18]. In the mammary gland of non-lactating heifers and lactating cows, both proximal (−1.4 kb down to −700 nt) and distal (−10.4 kb down to −9.9 kb) upstream regulatory regions of the bovine *CSN1S1* gene have been shown to be methylated [4], [12] to lower levels, than the liver which will never express the gene. Furthermore, inflammation induced by mastitis has been shown to reduce *CSN1S1* expression and increase the DNA methylation of three CpG located within a distal upstream regulatory region of the gene (Figure 1B and 1C) [4].

We therefore hypothesized that ODM might induce long-term effects on milk production by altering the DNA methylation profiles around genes which play key roles in milk production. As a first step, we studied the variations induced by unilateral ODM (for one week) with respect to milk production and the accumulation of *CSN* transcripts in the mammary tissue. We then analysed the methylation profiles of four CpG sites located in the regulatory region of the *CSN1S1* gene. Three had already been shown to be modified in the event of inflammation or during involution [4], [5] but a fourth CpG sequence had not previously been studied. It is located within a potential binding site) for STAT5, a key transcription factor within complexes regulating milk protein gene expression [19]. We also describe for the first time that this regulatory region is upstream from an RNA transcribed in the mammary gland that we detected by RNA-Seq.

## Materials and Methods

### Ethics Statements

All animal experiments were carried out in compliance with French regulations on animal experimentation and with the authorization of the French Ministry of Agriculture (Animal Health and Protection Directorate, accreditation number C35-275-23). They also complied with the accepted standards of humane animal care.

### Herd enrolment, animals, milk and tissue samples

Eight primiparous heifers (Bos Taurus, Prim'Holstein) from INRA's experimental farm at Mejusseaume (INRA, IEPL, Le Rheu, France) were studied just after peak lactation between 53 and 74 days in milk (DIM), when milk production is expected to be almost stable. Primiparous cows were chosen for two reasons. The relative decrease in milk yield after ODM is generally larger than in multiparous ones [20]. Furthermore, primiparous cows will not exhibit carry-over effects from undetected mastitis or any other events that might have occurred during previous lactation and induced long term effect on milk production. Cows with milk production well-balanced between their two udder halves were selected, taking care to avoid animals displaying any teat damage or non-functional quarters. Among them, six heifers had delivered at the age of 2.0±0.1 years and two at the age of 2.9+0.05 years. None of the cows were inseminated post partum and during the study period.

The cows were then moved to the experimental room one week before the start of the protocol so that they could adapt to this new environment. Throughout their housing in the experimental unit, they received the same diet as that given to the herd, except that they were not given any hay. They were fed ad libitum according to INRA guidelines with a diet containing 71.4% corn silage, 17% supplement, 10% soybean meal and 0.6% urea on a dry matter basis. They were milked twice daily from parturition to the start of the protocol.

The experimental protocol included three, one-week periods: P1, starting at 53.1±1.7 DIM, P2 and P3. During P1, (week 8 after parturition), both udder halves were milked twice a day for 7 days. On day 8, which was the first day of P2 (53+7 days after parturition), the right udder half was milked twice a day and the left one only once a day (in the morning) and this differential milking was continued for another 7-day period. Cows were then returned to twice daily milking (P3). During P1 and P2, milk production from each udder half was measured from day 1 to day 5 and from day 1 to day 8, respectively. Milk samples were collected and kept for further analyses. During P3, milk production was evaluated on days 3, 6, 7 and 8.

The mammary glands from four pregnant cows (day 60 of pregnancy) were collected at the slaughter house, just after death. For the preparation of nuclear extracts and EMSA, two rabbits were euthanised at day 28 of pregnancy and day 6 of lactation, less than one hour after morning nursing. Their mammary glands were dissected immediately and snap frozen in liquid nitrogen before long-term storage at −80°C.

### Milk analyses

Milk protein, fat and lactose contents were determined by a commercial laboratory using an infrared method (Lillab, Châteaugiron, France) in milk samples collected during the morning and/or afternoon milkings. The total Nitrogen (N, Kjeldahl) and non-casein N (soluble at pH 4.6 in the presence of 10% acetic acid and 1 M sodium acetate) contents were analysed in milk samples collected from each udder half at the morning milking on day 7 of each period. Casein contents were estimated as the total Nitrogen (N) minus soluble N at an acid pH relative to total N. An aliquot of each milk sample was skimmed after centrifugation at 1,500 g for 15 min at 4°C and then used for α-lactalbumin radial immunodiffusion (RID) analyses using the RID kit BOV Ala Test (ID Biotechnologies, Issoire, France). Skimmed milk samples were diluted at 1∶100 in SRID buffer (ID Biotechnologies) and loaded (15 µl/well) onto RID plates containing agar gel with anti-α-lactalbumin antiserum. The plates were incubated in a humidified atmosphere at 37°C for 48 h. The plates were then incubated with 2% acetic acid for 2 min and rinsed with water. Images were captured using the IDRing Viewer (ID Biotechnologies) and the precipitation rings were analysed with IDRing Meter software (ID Biotechnologies).

### Biopsies

On the day following P2 (i.e. the first day of P3), biopsies (7 cm long and 0.5 cm in diameter) were sampled before the morning milking. Biopsies were taken from the upper portion of the rear quarter of the mammary gland using a 70×4 mm instrument as previously described [2]. After surgery, the cows received antibiotic therapy by means of i.m. injections of 0.6 g Naxcel (PFIZER, Paris, France) for three days.

The biopsies were cut into pieces. Tissue specimens for RNA, DNA and protein nuclear extractions were snap frozen in liquid nitrogen before long term storage at −80°C until use, while those for histological analyses were fixed and processed as described below.

### Histology

For histology, the mammary specimens were fixed in RCL2 (Excilone, Elancourt, France) for 24 hours at 4°C. They were then dehydrated in 70% ethanol and embedded in paraffin according to standard histological protocols. Five micrometer sections, lying at least 100 mm apart, were mounted on slides. The slides were stained with Hematoxylin-Eosin-Safran (Sigma, Saint Quentin Fallavier, France) and then digitalized using a scannerNanoZoomer (Hamamatsu Photonics, Hamamatsu City, Japan). The latter technique enabled observation of the entire section. Five sections per cow were processed and the images were analysed using both the Hamamatsu NanoZoomer Digital Pathology Virtual Slide Viewer and Image J software (Wayne Rasband; National Institute of Health, Bethesda, MD).

### Electron microscopy

Specimens for transmission electron microscopic observations were fixed with 2% glutaraldehyde in 0.1 M sodium cacodylate buffer, pH 7.2, for 4 hr at room temperature, and then post-fixed with 1% osmium tetroxide containing 1.5% potassium cyanoferrate, gradually dehydrated in ethanol (30% to 100%), and embedded in Epon (Delta Microscopy, Ayguesvives, France).

Semi-thin sections (1µm) were collected onto glass slides, counterstained with methylene blue-Azur II and imaged on a Leica Leitz DMRB microscope equipped with a DP50 camera (Olympus) coupled to Cell software (Olympus). Thin sections (70 nm) were collected on 200-mesh copper grids, and counterstained with lead citrate before examination with a Zeiss EM902 electron microscope operating at 80 kV (Carl Zeiss, France). Images were acquired with a charge-coupled device camera (Megaview III) and analysed with ITEM Software (Eloïse, France) at the MIMA2 facilities (www.6.jouy.inra.fr/mima2, UMR1313 Génétique Animale et Biologie Intégrative, INRA, Plateau de Microscopie Electronique, F78350 Jouy-en-Josas, France).

### RNA extraction

Total RNA was extracted from a 50 mg specimen using 750 µl Trizol LS reagent (Invitrogen, Cat. N°. 10296-010), treated with DNase I (QIAGEN, Cat. N°. 79254) and then purified using NucleoSpin RNA Clean-up (Macherey-Nagel, Cat. N°. 740948.50). Total RNA was eluted with 50 µl DNase I-free water. Approximately 20 to 30 µg were recovered per biopsy.

RiboMinus RNA (ribosomal RNA-depleted RNA) was purified from 5 µg total RNA using the RiboMinus Eukaryote Kit for RNA-Seq (Invitrogen, Cat. N°. A10837-08).

RNA Integrity was assessed using an Agilent Bioanalyzer.

### RNA sequencing

Four RNA samples per tissue specimen at each stage or for each treatment were analysed. They came either from the udder halves of lactating cows after ODM or from the contralateral glands after TDM, or from the mammary gland of pregnant cows (day 60 of pregnancy). RiboMinus RNA (100 ng) from each biopsy were prepared, pooled two by two and outsourced for deep RNA sequencing to IMAGIF. RNA were fragmented using RNA fragmentation reagents (Ambion) according to the manufacturer's recommendations. They were then treated with antarctic phosphatase (NEB) and Polynucleotide Kinase (NEB). Strand orientated libraries were prepared with the Truseq Small RNA sample prep Kit (Illumina), the final gel purification step being replaced by a PCR cleanup with AMPureXP beads (Beckman-Coulter).

40 bp single reads were generated on an Illumina Genome Analyzer IIx, using the TruSeq SR Cluster Kit v2-cBot-GA and TruSeq SBS Kit v5.

Read quality was checked with FastQC in the ng6.toulouse.inra.fr environment [21]. The reads were then spliced-aligned to the bovine reference genome (UMD3.1) using the TopHat v2.0.5 software package [22]. The resulting bam files were processed with a modified version of CUFFLINKS 2.0.0 [23], called sigcufflinks, which also produces raw read counts per transcript (available upon request at sigenae.org), in order to discover genes and quantify transcripts. The quantification results were merged in the expression table. The merged GTF file was also used to extract the fasta sequence of the newly discovered transcripts so that they could be annotated.

The 39 bp reads thus generated were mapped on the Bos_taurus.UMD3.1.65 genome using IGV2.3 software from the Broad Institute. RNA-Seq data have been deposited on the Annotare website http://www.ebi.ac.uk/fg/annotare/; under accession number E-MTAB-2755.

### RT-qPCR

Total RNA (625 ng) was reverse transcribed as already described [2], using p(dT) (Roche) or specific primers described in Table 1, and amplified using the Absolute Blue SYBR green Rox Mix from Thermo Scientific. The primers used to amplify CSN mRNA were those already described [24]. Primers used to amplify RNA transcribed from the distal upstream region are described in Table 1. The forward and reverse primers used to amplify the reference cyclophilin A-like mRNA were 5′- GGTGACTTCACACGCCATAATGGTAA-3′ and 5′-GGACAAGATGCCAGGACCTGTATG-3′, respectively.

**Table 1:** Primers used to analyse RNA transcribed from the CSN1S1 upstream region.

AS-RT-P1	5′-CACGTGCTCTGAGCT-3′
AS-RT-P2	5′-AATGAGGCCAAGTCA-3′
AS-RT-P3	5′-TCCACATCACTATCA-3′
AS-RT-P4	5′-CCTGCCAAATCCTCA-3′
S-RT-P1	5′-CTTTGTTATAAAGGACT-3′
S-RT-P2	5′-CAAAGTCTTAGCCACT-3′
S-RT-P3	5′-CAAGCCCCCTGCAGT-3′
S-RT-P4	5′-CTTTAGTTTGTGACAT-3′
ASPR2	5′-CCACATCACTATCAGCCATGTCAGCT-3′
ASPF1	5′-GTTATAAAGGACTTTATTGGGACAACTGGA-3′

After amplification, representative samples and the 100bp DNA ladder from Biolabs diluted or not in qPCR buffer (L+ and L-, respectively) were loaded on 2% agarose gels stained with ethidium bromide.

### Genomic DNA extraction

Genomic DNA (gDNA) was extracted from mammary specimens (50 mg) as previously described (Molecular Cloning – Laboratory Manual, Sambrook and Russell, 2001). The purity and quantity of gDNA were checked on agarose and using the Nanodrop ND-1000 (Labtech).

### Bisulfite treatment, amplification and pyrosequencing of the *CSN1S1* regulatory region

Bisulfite pyrosequencing was performed as described previously [25]. Briefly, one µg of the EcoRI-linearised gDNA was incubated in the dark in 0.5 ml freshly prepared sodium bisulphite 5 M and hydroquinone 130 mM at 55°C for 4 hours. Bisulfite treated gDNA was then purified using the Wizard DNA clean-up system (Promega, Cat. N°. A7280), incubated in 0.3M NaOH at 37°C for 20 min and then ethanol precipitated overnight at −20°C in the presence of 2M ammonium acetate. Precipitated DNA was recovered after 45 min at 14,000 g and the pellet was dissolved in 20 µl water. One µl of bisulfite-modified gDNA was then amplified using a pair of primers of which one was biotinylated in order to capture the antisense DNA strand for further pyrosequencing. PCR reactions were carried out in 25 µl reactions containing 2 mM MgCl_2_, 0.4 µM of each primer (Table 2), 0.4 mM dNTPs, and 2 IU Platinum Taq DNA Polymerase (Invitrogen, Cat. N°. 10966-034). The cycling conditions were as follows: Initial denaturation at 95°C for 5 minutes; 45 cycles including: denaturation at 95°C for 30 seconds, annealing at 54°C for 30 seconds, elongation at 72°C for 30 seconds; an extension step at 72°C for 5 minutes.

**Table 2:** Primers used to amplify the CSN1S1 upstream region after bisulfite conversion or to prime the pyrosequencing reactions.

PCR Forward 1	5′-ATAGTTTGTATTGTTTTTAAAAGATTATGGT-3′
PCR Reverse 1	5′-ATTAATAAAACCCTCCCTATTCCAAC -3′
PCR Forward 2	5′-GATTTGAAATAGCAAGAGGAAGTTATGA-3′
PCR Reverse 2	5′-CAAACAATACAAAAAAAAATTTAAACTTAATTCT-3′
Sequencing CpG1	5′-GATTATGGTTTTATTTGA-3′
Sequencing CpG2	5′-TTTGTTTTTTTAGGTGA-3′
Sequencing CpG3	5′-GTTTAGAAATAAAGGA-3′
Sequencing CpG4	5′GTTTTTTAGGTATTTGTTT-3′

The sequences of the primers were designed to take account of the fact that after bisulfite conversion all non methylated C have been converted to T and that this conversion induced the presence of A on the complementary and reverse strand. Both T and A resulting from the bisulfite conversion of C are underlined.

The antisense DNA strand was captured using Streptavidin Sepharose High Performance (GE-Healthcare, Cat. N°. 17-5113-01), hybridised with sequencing primers and run on a Pyrosequencer (PyroMarkQ24, QIAGEN). The percentage of DNA methylation at each CpG was analysed with the software provided by QIAGEN. PCR and sequencing primers were designed using Methyl Primer Express v1.0 software. Their sequences are described in Figure 1C and Table 2.

### Cloning and sequencing

DNA fragments amplified as described above for pyrosequencing were cloned in TOPO TA Cloning vector (TOPO TA Cloning kit, pCR2.1-TOPO vector Invitrogen 45-0641) and the inserts sequenced by GATC Biotech SARL, France.

### Preparation of nuclear extracts

The preparations of mammary gland nuclear extracts or total cellular extracts were carried out as previously described [26]. STAT5 has been shown to be only transiently activated, within an hour, by PRL. Therefore, to prepare activated STAT5, mammary biopsies should have been sampled just after milking (whereas in our protocols, biopsies were samples before milking). As an alternative, we took advantage of the fact that rabbit dams nurse their pups once a day, in the early morning, that STAT5 and STAT5 binding elements are conserved between species [26]–[29] and decided to collect rabbit mammary tissue at the slaughter house just after morning suckling.

### Electrophoretic Mobility Shift Assay (EMSA)

EMSA was performed as previously described [26] using labelled double strand oligonucleotides mixed or not with double strand unlabelled oligonucleotides (competitors).

The sense oligonucleotides described in Table 3 (50 ng) were 5′ end labelled with ^32^P. Labelled sense oligonucleotides (50 ng) were then annealed with a 6-fold amount of unlabelled antisense oligonucleotides in 20 µl and double strand labelled probes were purified on non denaturating 12% polyacrylamide gels in 1xTBE.

**Table 3:** Description of sense probes used in EMSA.

Probe	Sequence
C	5′-GAGAATTCTTAGAATTTAAA-3′
D1	5′-CTGCCTTCCTCGAAGGAAAA-3′
D2	5′-TCTAATTCTCAGAATCAAGT-3′
D1m	5′-CTGCCTTCCTC^m^GAAGGAAAA-3′

The sequence of the C probe located between −103 and −84 nt upstream from the transcription start site of the bovine CSN1S1 gene is identical to that of the functional STAT5 binding site found in the promoter region of the rabbit CSN1S1 gene [63].

For competition binding assays, double strand unlabelled oligonucleotides were prepared as follows. Sense and antisense oligonucleotides (3 µg each) were annealed by heating at 95°C for 5 min and then slowly cooled to room temperature in a total volume of 15 µl containing 10 mM Hepes pH 7.4, 1 mM EDTA, 50 mM NaCl. The completeness of annealing and the concentration of double strand oligonucleotides were evaluated by comparison with the GeneRuler 50 bp DNA Ladder (Thermo Scientific Life Science Research #SM0373) on a nondenaturing 12% polyacrylamide gel in 1x TBE. These stock solutions were then kept at −80°C. Serial 2-fold dilutions of the stock solutions in annealing buffer (10 mM Hepes pH 7.4, 0.1 mM EDTA, 50 mM NaCl) were prepared. A constant amount of double strand labelled probe (C, 555 pg or D2, 550 pg) was finally mixed with increasing amounts of the unlabelled double strand competitor (0–200 ng) in a total volume of 10 µl.

Rabbit mammary gland nuclear extracts (22 µg protein) were incubated with the indicated labelled double strand probes (60,000 cpm/reaction), mixed or not with increasing amounts of the competitor, for 30 minutes, in a total volume of 10 µl. In the absence of a competitor, 183 pg, 269 pg, 245 pg, 220 pg were introduced for the C, D1, D1m and D2 oligonucleotides, respectively; corresponding to 15–22 fmol, whereas in the presence of a competitor 555 p and 550 pg were used for the C and D2 probes, respectively.

For supershift experiments, mouse STAT5a or STAT5b antibodies (1 or 0.5 µg; Invitrogen 13-3600 and 13-5300, respectively), which cross-react with rabbit STAT5 [26], were then added and the reaction was continued for another 30 minutes.

Complexes were analysed on non denaturating 5% or 6% polyacrylamide gels in 0.25x TBE. The migrations were visualized and signals corresponding to shifted complexes were quantified using the STORM 860 Phosphoimager (Molecular dynamics). Competition binding curves were fitted using the 4-parameter variable slope of Graph Pad software. Each experiment was repeated at least three times.

### Statistical analyses

Milk data obtained during the differential milking period and during the twice daily milking switchback period were analysed separately using the same statistical model. The average daily milk yield per udder half and milk composition data were analysed by ANOVA using the SAS MIXED procedure (SAS Institute, 1999). The data obtained during the pretreatment period were averaged and used as covariate per udder half. The effects of milking frequency (once or twice daily) and cow were tested. The averaged milk data and composition obtained during the pretreatment period were compared between udder halves using a paired Student's t-test.

Statistical analysis to determine the differential accumulation of transcripts was performed using the R software version 3.0.0 (R Development Core Team, 2013) and the Bioconductor package DESeq2 version 1.0.0 [30]. DESEQ2 utilizes a negative binomial distribution to model read counts per mRNA and implements a method to normalise the counts. This normalisation procedure uses the library median of the ratios between the read count and the geometric mean of each gene as a scaling factor for each library. Fold changes were estimated with an empirical Bayes shrinkage procedure. This helps to moderate the broad spread in fold changes for genes with low counts, while it has negligible effects on genes with high counts [30].

The test for differential transcript levels was only performed with respect to genes for which the sum of counts across all four samples counts was higher than 50 (16864/22715 genes). The p-values were adjusted for multiple testing using the Benjamini and Hochberg method [31]. Differences with an adjusted p-value (q-value) <0.1 were considered to be significant.

None of the CpG methylation levels displayed a Gaussian distribution in any of the sample types. A Wilcoxon paired test was therefore used to evaluate differences between ODM and TDM samples for each CpG and a Wilcoxon Mann-Whitney test used for other comparisons. An analysis of variance and the Tukey's HSD test [32] were used to evaluate the significance of the differences in the methylation levels of CpG. These analyses were performed with R software version 3.0.0 (R Development Core Team, 2013).

## Results

Eight Holstein cows were milked bilaterally twice daily from the onset of lactation to the peak of lactation (around 51 days in milk). Their daily milk yield per udder half during one week (P1) was then precisely measured and the milk composition analysed. These analyses were pursued for the next two week-periods, P2 and P3.

### Milk yield decreased with ODM and remained lower even when TDM was resumed

**Figure 2:**
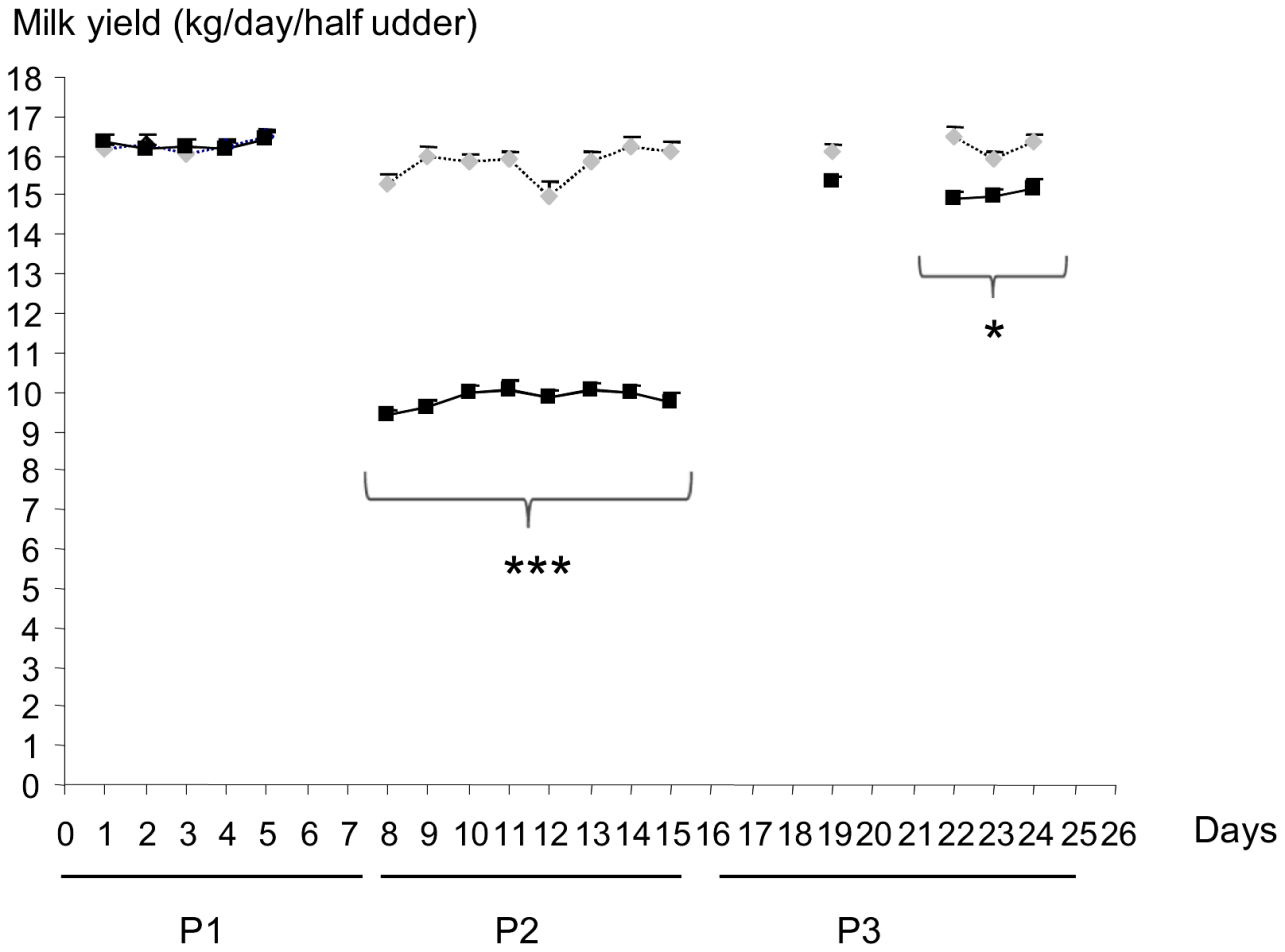
Average milk yield for each udder half of the eight cows during the three periods. Milk yield from the left right udder is indicated by black squares, whereas that of the left one is indicated by grey diamonds. The three periods are: P1, twice daily milking of both udder halves; P2, twice daily milking of the right udder half and once daily milking of the left one; P3, twice daily milking of both udder halves. Significant differences between the amounts of milk produced by the two udder halves are indicated by stars: *** P<0.001; * P<0.05.

During P1, the average milk yield did not differ between udder halves (16.3±0.17 kg/day, p-value  = 0.97) (Table 4). During the first 24 hours of P2, unilateral ODM of the left udder halves induced a sharp decrease in milk yield to an average value for the eight cows of 9.4 kg/day (Figure 2). The average milk yield during P2 (9.8 kg/day) remained lower than that of the contralateral udder (15.8 kg/day), exhibiting a significant decrease (P<0.001) which varied from 4.8 to 7.6 kg/day depending on the animal (Table 5).

**Table 4:** Average daily milk yield and milk composition during P1.

Udder half	Units	Right	Left	Differences		
Milking frequency		TDM	TDM	Right - Left	SEM	p-value
Daily milk yield	kg/day	16.3	16.3	0	0.33	0.97
Daily fat yield	g/day	545	545	0	19.4	0.99
Milk fat content	g/kg	33.4	33.5	−0.1	0.85	0.69
Daily protein yield	g/day	440	441	−1	10.9	0.93
Milk protein content	g/kg	27.1	27.1	0	0.36	0.77
Daily casein yield	g/day	343	342	1	7.7	0.88
Milk casein content	g/kg	21.1	21.1	0	0.29	0.75
Milk α-lactalbumin content	g/kg	0.98	0.99	−0.01	0.03	0.57
Lactose yield	g/day	828	828	0	17.1	0.99
Lactose content	g/kg	51.0	50.9	0.1	0.51	0.67

All contents are expressed relative to kg of milk, except α-lactalbumin contents which are expressed relative to kg of skimmed milk. The averaged milk data (n = 8) were compared between udder halves using a paired Student's t-test.

**Table 5:** Average daily milk yield and milk composition during P2.

Udder half	Units	Right	Left	Differences		
Milking frequency		TDM	ODM	TDM-ODM	SEM	p-value
Daily milk yield	kg/day	15.8	9.8	6.0	0.22	<0.001
Daily fat yield	g/day	510	399	111	12.5	<0.001
Milk fat content	g/kg	32.2	40.8	−8.6	0.93	<0.001
Daily protein yield	g/day	414	260	154	10.4	<0.001
Milk protein content	g/kg	26.2	26.5	−0.3	0.28	0.48
Daily casein yield	g/day	340	204	136	8.1	<0.001
Milk casein content	g/kg	21.5	20.8	0.7	0.21	0.04
Milk α-lactalbumin content	g/kg	0.94	0.81	0.13	0.02	0.01
Lactose yield	g/day	790	489	301	9.8	<0.001
Lactose content	g/kg	50.2	49.7	0.5	0.15	0.08

All contents are expressed relative to kg of milk, except α-lactalbumin contents which are expressed relative to kg of skimmed milk. Differences between milk data (n = 8) were evaluated using the SAS MIXED procedure with P1 data as the covariable.

Three days after the return to bilateral twice daily milking, the milk yield from left udder halves increased in all eight cows (Figure 2). During P3, the milk yield reached an average value of 15.1±0.2 kg/day, which was close to but lower than that observed in the contralateral glands (16.2±0.2 kg/day) that had always been milked twice daily (P<0.001, Table 6). In 7 out of the 8 cows, the milk yield did not reach the values observed in the contralateral glands (data not shown). This lower milk yield in the half udder milked once daily over P2 was not due to a global reduction in milk production as no significant differences throughout P1 to P3 are observed in the right udder halves which had always been milked twice daily (Table 4–6 and Figure 2).

**Table 6:** Average daily milk yield and milk composition during P3.

Udder half	Units	Right	Left	Differences		
Milking frequency		TDM	ODM	TDM-ODM	SEM	p-value
Daily milk yield	kg/day	16.2	15.1	1.1	0.07	<0.001
Daily fat yield	g/day	568	529	38	7	0.01
Milk fat content	g/kg	35.0	35.1	−0.1	0.32	0.78
Daily protein yield	g/day	455	434	21	3.9	0.01
Milk protein content	g/kg	28.0	28.7	−0.7	0.07	<0.001
Daily casein yield	g/day	351	337	14	2.3	<0.01
Milk casein content	g/kg	21.7	22.3	−0.6	0.14	0.02
Milk α-lactalbumin content	g/kg	0.88	0.86	0.02	0.02	0.47
Lactose yield	g/day	822	765	57	4.4	<0.001
Lactose content	g/kg	50.8	50.8	0	0.05	0.86

All contents are expressed relative to kg of milk, except α-lactalbumin contents which are expressed relative to kg of skimmed milk. Differences between milk data (n = 8) were evaluated using the SAS MIXED procedure using P1 data as the covariable.

These variations in milk yield during ODM were probably associated with variations in milk composition. The milk protein composition was therefore studied.

### Milk composition varied during and after ODM

The daily protein yield was similar between the two udder halves during P1. However, during differential milking, it varied both between the three periods and between udder halves (Table 4–6). During P2, the daily protein yield from the left udder halves milked once daily was significantly lower by 154±10.4 g/day (37.1%, P<0.001) than that observed in the milk of the contralateral udder halves which had always been milked twice daily (Table 5). During P3, it was still lower by 21±3.9 g/day (4.6%, P<0.01).

The milk protein content, was similar between both udder halves during P1 and P2 (p-values  = 0.77 and 0.48, respectively). However, during P3, the milk protein content was slightly higher (2.5%) in the udder halves that were switched back to TDM by comparison with those that had always been milked twice daily (28.7 vs 28.0±0.07 g/kg, P<0.001, Table 6).

Among these milk proteins, CSN represent the largest fraction. Daily CSN yields were similar between the two udder halves, during P1. During P2, these levels in the left udder halves milked once daily were lower by 136±8.1 g/day (40.1%, P<0.001) than those observed in the milk of the contralateral udder halves (Table 5). During P3, they were still lower by 14±2.3 g/day (4.0%, P<0.01). Throughout P1 to P3, variations in daily CSN yields therefore wholly reflected those of the daily protein yields. By contrast, variations in the milk CSN content during P2 were observed in the absence of any significant change to the milk protein content. The milk CSN content from the left udder halves milked once daily was slightly lower than that observed in the milk from contralateral udder halves (20.8 vs 21.5±0.2 g/kg (3.2%, p-value = 0.04, Table 5). However, during P3, the variation in milk CSN content paralleled that of the milk protein content. The milk CSN content was higher in udder halves that were switched back to twice daily milking when compared to those that had always been milked twice daily (22.3 vs 21.7±0.1 g/kg (2.8%, p-value  = 0.02, Table 6). These different variations between milk or casein yields and casein contents were not surprising as no quantitative and simple relationships exist between these parameters. The mechanisms that control how casein concentrations drive the casein content in milk involve a series of steps which include the supramolecular assembly of caseins and synchronized secretion processes of whole milk products. The change affecting these mechanisms after ODM still need to be studied.

α-lactalbumin, a minor component of bovine milk, is involved in lactose synthesis and plays a major role in milk production. Variations in milk α-lactalbumin contents were similar to those affecting CSN during P2. During P3, milk from both udder halves had similar α-lactalbumin contents. These variations in milk synthesis were in line with the variations observed in lactose contents (Table 4–6). Milk fat yield also clearly decreased with ODM, and remained significantly lower when TDM is resumed whereas milk fat content significantly increased with ODM but was no longer altered when TDM was resumed.

The variations in milk yield and composition after one week of ODM suggest that ODM decreases the volume of milk produced and also slightly lowers its casein and α-lactalbumin concentrations. They may result from modifications to mammary tissue structure. The structure of the mammary tissue in udder halves milked once or twice daily was therefore studied on biopsies collected at the end of P2.

### Histological and ultrastructural analyses of mammary biopsies

**Figure 3:**
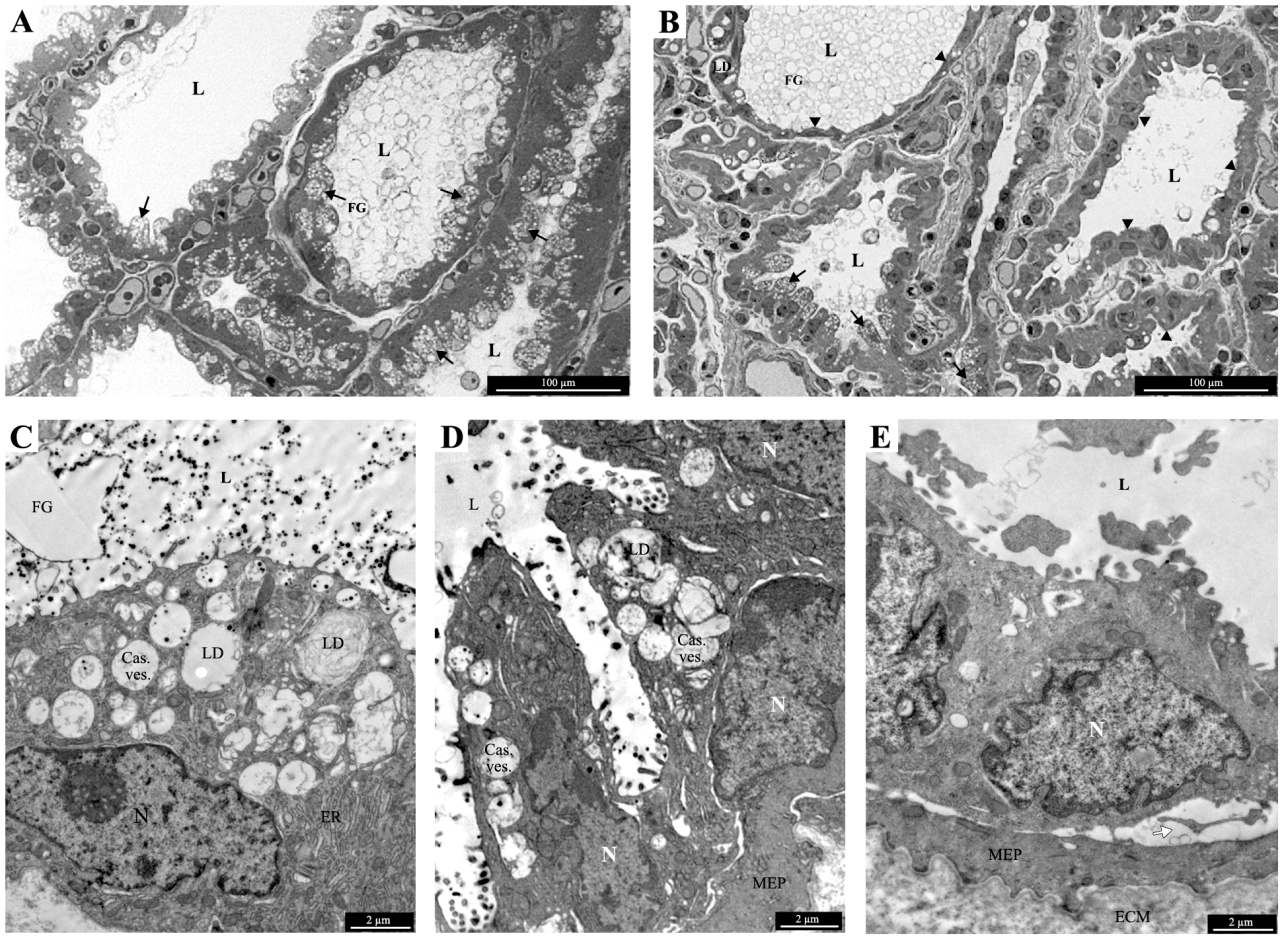
Morphological analyses of mammary biopsies. Semi-thin (1 µm) and thin sections (70 nm) of biopsies were observed using optical microscopy (A and B) or Transmission Electron Microscopy (C to E), respectively. Sections were obtained from mammary glands milked twice daily (A and C) or once daily (B, D and E). L: lumen, *: secretion vesicles, FG: fat globule, Cas. Ves: casein vesicles, ER: endoplasmic, MEP: myoepithelial cell, N: nucleus, LD: lipid droplet, ECM: extracellular matrix. White arrows indicate the intercellular space, black arrows indicate cells with secretion vesicles and black arrowheads indicate cells without secretion vesicles.

Mammary biopsies were collected before morning milking. Sections (5 µm) observed using the NanoZoomer technology showed that mammary epithelial tissue represented 70±10.1% and 69+14.9% of the biopsies after TDM and ODM, respectively. The biopsy content in epithelial tissue was not altered by the milking frequency. Furthermore, no clear histological differences could be observed between udders milked once or twice daily. However, thinner tissue sections (1 µm), which were prepared for observation at higher magnifications using optical or transmission electron microscopy, exhibited differences between the two udder halves of three cows out of five. After ODM, cells in some acini contained no secretion vesicles when observed at an optical scale (Figure 3), and they displayed a dense cytoplasm with only a few lipid droplets and no caseins vesicles, when observed by transmission electron microscopy (Figure 3). Such acini were not observed in sections from TDM specimens (Figure 3A). Their proportion after ODM ranged from 23% to 50%. The other type of acini had lumen which were filled with fat globules and casein micelles, which form typical structures that are easy to recognize using electron microscopy. The epithelial cells exhibited a large cytoplasm with lipid droplets and vesicles containing immature micelles and mature casein micelles clearly displaying active mammary synthesis and secretion (Figure 3B and D). These acini were similar to those observed after TDM (Figure 3A and 3C). ODM therefore induced heterogeneous ultrastructures of the different acini forming the mammary epithelium which could be related to impaired milk protein synthesis in some epithelial cells.

Mammary gland *CSN* mRNA profiles between udder halves milked once or twice daily were thus established.

### *CSN1S1*, *CSN2* and *CSN1S2* RNA profiles varied after ODM

**Figure 4:**
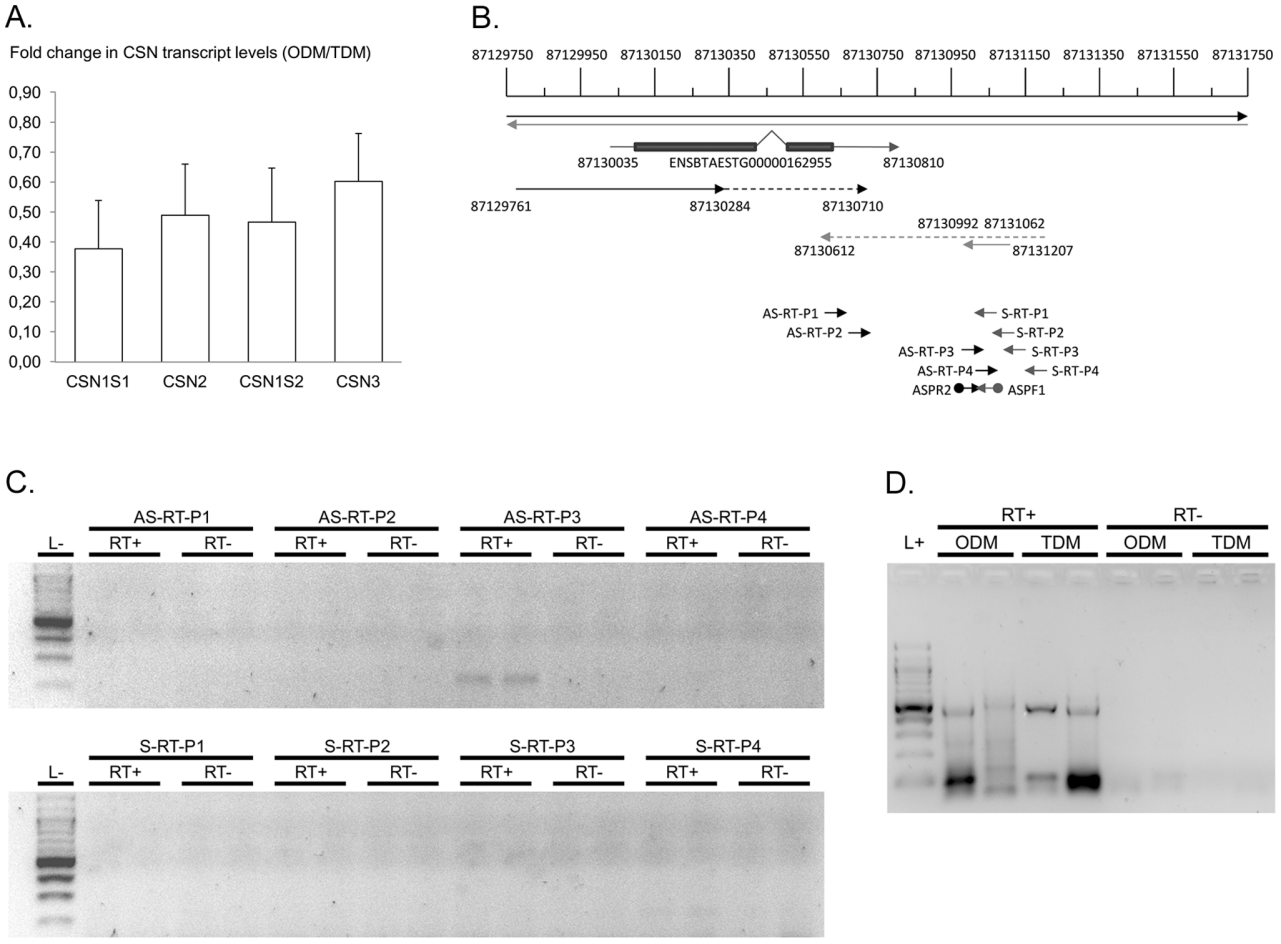
Analysis of transcripts in the *CSN* gene cluster. **(A)** Fold changes in CSN transcript levels after ODM when compared to TDM. Mean values of fold changes for each CSN transcript are shown as boxes and s.e.m. indicated as bars. **(B)** Mapping of RNA-Seq reads close to the distal regulatory region. RNA-Seq reads in RNA depleted of ribosomal RNA were mapped onto the bovine genome sequence. The positions on BT6 are indicated. Regions covered by sense reads are indicated by black arrows, while those covered by antisense reads are in grey. Regions for which reads were found in all cows are indicated by solid lines, while those only found in two or three animals are indicated by broken lines. The 5′ and 3′ untranslated regions of ENSBTAESTG00000162955 (already described in Ensembl release 75, February 2014) are indicated by blue solid lines, the coding regions of the two exons are boxed and the intron is indicated in between. Primers used for RT (arrows) and PCR (anchored arrows) are indicated in black or grey for sense and antisense orientations, respectively. **(C)** Mapping of RNA transcribed in the distal regulatory region of the *CSN1S1* gene. Total RNA extracted from the mammary gland of a lactating cow (99 DIM) were reverse transcribed using different primers in the sense or antisense directions (S-RT or AS-RT, respectively). Similar reactions were conducted in parallel in the absence of reverse transcriptase to monitor DNA contamination (RT-). All RT products were then amplified using ASPF1 and ASPR2 primers. Amplification products and a 100 bp DNA ladder (L-) were analysed on 2% agarose gels stained with ethidium bromide. **(D)** RT-PCR analysis of the antisense RNA transcribed in the distal region of the *CSN1S1* gene after ODM or TDM of two cows. Ribominus RNA were reverse transcribed (RT+) using AS-RT-P3. Similar reactions were conducted in parallel in the absence of reverse transcriptase to monitor DNA contamination (RT-). RT+ and RT- products were then amplified using ASPF1 and ASPR2 primers. Amplification products and a 100 bp DNA ladder in RT-PCR buffer (L+) were analysed on 2% agarose gels stained with ethidium bromide.

The concentrations of the different *CSN* mRNA in total RNA were evaluated by RT-qPCR (Table 7). An average decrease of 62% in *CSN1S1* (P = 0.05) was observed in the eight cows after ODM, when compared to TDM. This reduction ranged from 83% to 100% for four cows but was limited between 12% and 57% for the other four. CSN2, CSN1S2 and CSN3 transcripts exhibited similar variations both between ODM and TDM (P-Values  = 0.08, 0.07 and 0.07, respectively) and between animals, as is clearly observed by the bar plot (Figure 4A). Transcripts from the *CSN* cluster were studied further by RNA-Seq on specimens from four cows, two exhibiting extreme variations (12% and 100%) and two exhibiting moderate variations (21% and 57%) in CSN1S1 levels after ODM. Two pools of samples were prepared: one which included a sample exhibiting a marked variation and a sample exhibiting a low variation, and the other which included two samples with moderate variations. RNA-Seq analyses revealed that *CSN* transcript accumulation in the mammary gland reached very high levels during lactation in TDM udder halves (Table 8), when compared to reference transcripts such as RPS9 and RPS15 [33]. Their levels were at least 100 fold higher than at day 60 of pregnancy (Table 9 and 10). Among these *CSN* transcripts, *CSN1S1* and *CSN3* mRNA reached the highest levels during lactation. In the four lactating cows selected for RNA-Seq analyses, ODM induced a two-fold fall in *CSN2* transcript levels which paralleled that of alpha-lactalbumin (*LALBA*), whereas the reference transcripts (*RPS9* and *RPS15*) were not modified (Table 8). This RNA-Seq approach limited to four animals revealed a two-fold reduction in the levels of all other *CSN* transcripts (CSN1S1, CSN1S2 and CSN3; Table 8) with q-values ranging from 0.06 to 0.096. These results are in agreement with those obtained by RT-qPCR. RNA-Seq data further revealed that all *CSN1S1* transcripts in the bovine mammary gland during lactation did not share the same 5′ends. Some 5′ends were located around BT6: 87.141.556, as described for the *CSN1S1* transcriptional start point in Ensembl release 75, February 2014, others were located further downstream, whereas most transcripts were initiated at position 87.141.591, 24 nucleotides away from a classical TATA box, as previously described [34]. In this work we used that site as a reference when describing the *CSN1S1* regulatory region.

**Table 7:** Evaluation of transcripts in the bovine mammary gland during lactation after ODM or TDM using RT-qPCR analyses.

Gene symbol	ODM	TDM	ODM/TDM			
	ΔCt	ΔCt	-ΔΔCt	Fold change	s.e.m.	p-value
CSN1S1	−5.261	−9.023	−3.761	0.38	0.13	0.05
CSN2	−5.223	−8.544	−3.321	0.49	0.17	0.08
CSN1S2	−3.814	−7.215	−3.401	0.47	0.18	0.07
CSN3	−5.730	−7.605	−1.875	0.60	0.16	0.07

CSN transcripts in total RNA (1.25 µg) were evaluated using RT-qPCR. Data were normalized relative to CCPL transcript (ΔCt). Differences between ODM and TDM were evaluated as ΔΔCt and fold changes calculated. Differences were estimated by a paired t-test.

**Table 8:** Evaluation of transcripts in the bovine mammary gland during lactation after ODM or TDM using RNA-Seq analyses.

Gene symbol	Gene Id	ODM	TDM	ODM/TDM	q-value
CSN1S1	ENSBTAG00000007695	371831	750245	0.52	0.096
CSN2	ENSBTAG00000002632	193841	448346	0.48	0.029
HSTN	ENSBTAG00000048250	3021	6642	0.46	0.013
STATH	ENSBTAG00000024683	42	67	0.65	0.581
CSN1S2	ENSBTAG00000005005	162642	392410	0.48	0.065
ODAM	ENSBTAG00000006810	9	49	0.44	0.090
CSN3	ENSBTAG00000039787	304260	602687	0.52	0.078
LALBA	ENSBTAG00000005859	135456	357379	0.43	0.019
RPS9	ENSBTAG00000006487	1646	1939	NS	0.879
RPS15	ENSBTAG00000019718	1199	1330	NS	0.969

Transcripts were evaluated by RNA-Seq. The ODM and TDM data were normalised, the fold change evaluated between paired values of ODM and TDM (ODM/TDM), and the adjusted p-value (q-value) was analysed as described in Materials and Methods.

**Table 9:** Evaluation of transcripts in the bovine mammary gland during pregnancy using RT-qPCR analyses.

Gene symbol	Pregnancy	Lactation	Ratio		
	ΔCt	ΔCt	-ΔΔCt	Fold change	p-value
CSN1S1	4.128	−9.023	13.15	0.001	<0.001
CSN2	3.035	−8.544	11.58	0.005	<0.001
CSN1S2	8.955	−7.215	16.17	0.010	<0.001
CSN3	−0.672	−7.605	−6.93	0.001	<0.001

CSN transcripts in total RNA (1.25 µg) were evaluated using RT-qPCR. Data were normalized relative to CCPL transcript (ΔCt). Differences between pregnancy and lactation were evaluated as ΔΔCt and fold changes calculated. Differences were estimated by a Wilcoxon Mann-Whitney test.

**Table 10:** Evaluation of transcripts in the bovine mammary gland during pregnancy using RNA-Seq analyses.

Gene symbol	Gene Id	Pregnancy	Lactation	Ratio	q-value
CSN1S1	ENSBTAG00000007695	1483	698851	0.002	<0.001
CSN2	ENSBTAG00000002632	1599	400472	0.004	<0.001
HSTN	ENSBTAG00000048250	9.7	6017	0.002	<0.001
STATH	ENSBTAG00000024683	0.3	68	0.005	<0.001
CSN1S2	ENSBTAG00000005005	51.8	3346215	0.000	<0.001
CSN3	ENSBTAG00000039787	3121	546873	0.006	<0.001
ODAM	ENSBTAG00000006810	ND			
LALBA	ENSBTAG00000005859	166	307315	0.001	<0.001
RPS9	ENSBTAG00000006487	1828	1573	1.162	0.553
RPS15	ENSBTAG00000019718	2096	2231	0.940	0.747

Transcripts were evaluated in ribominus RNA by RNA-Seq. Data were normalised, the fold change evaluated and the adjusted p-value (q-value) was analysed as described in Materials and Methods.

### The *HSTN* transcript from the *CSN* locus also varied after ODM

RNA-Seq further revealed that the accumulation of another transcript from the *CSN* cluster, *HSTN*, clearly decreased with ODM (q-value  = 0.01), whereas that of the *ODAM* transcript, expressed at lower levels, decreased but displayed quite a high q-value (0.09) in the four cows selected for this analysis. Although expressed at similar levels, when compared to *ODAM*, *STATH* was not affected (Table 8). Taken together, these results show that ODM does not only alter the expression of the *CSN* genes within the *CSN* cluster.

### RNA are transcribed upstream from the *CSN1S1* distal regulatory region

RNA-Seq data from strand orientated libraries also revealed that reads were found in the distal upstream region of the *CSN1S1* gene (Figure 4B). Some of these reads were of the sense type, while others were antisense.

In the former case, in all the mammary specimens analysed, they were encoded from BT6 87.129.761 to 87.130.284, and in some cows this extended further downstream to 87.130.710 (16 to 34 reads in each mammary specimen analysed). Part of the sequence in the region covered by these sense reads (Figure 4B, from 87.129.768 to 87.130.451) exhibited clear homology with part of the coding region (exon 15 to part of exon 21) of the Bos Taurus nuclear VCP-like mRNA, a transcript variant 1 (NVL) or transcript variant 2 for which the genes are located on BT16. These sense reads may represent a VCP- like pseudogene. Another part of the sequence covered by the sense reads (from 87.130.035 to 87.130.417) has been already described as ENSBTAESTT00000162955. This transcript has a 155 amino acid open reading frame.

Surprisingly, some sense reads (from 87.130.612 to 87.130.710) were inverse and complementary to antisense reads (6 to 13 reads per sample analysed). Antisense reads could also be mapped further downstream. In all RNA samples, it was possible to map them between positions 87,130,992 and 87,131,062 (Figure 4B). In some RNA samples, this 70 nt region extended to position 87,131,207. These antisense reads were not detected in mammary RNA samples from four independent pregnant cows (data not shown) or in other reproductive tissues such as the ovary or testes (M. Pannetier, personal communication). This transcript was only amplified after RT-PCR with AS-RT-P3 (Table 1 and Figure 4B and 4C), as predicted by RNA-Seq (correct orientation and location). The signals varied considerably between animals, and the differences between ODM or TDM were not significant (Figure 4D). The antisense reads exhibited several stop codons on the three different translation frames. This transcript is therefore unlikely to be a coding RNA and it may be a long non-coding RNA.

No differences in the accumulation of either the sense or antisense transcripts were observed between ODM and TDM.

*CSN1S1* gene expression had previously been linked to the methylation profile of a distal upstream region of the *CSN1S1* gene after mastitis [4]. We therefore studied the methylation level of this regulatory region in more detail after ODM.

### Three CpG within a distal regulatory region of the *CSN* gene cluster were methylated to higher levels after ODM

**Figure 5:**
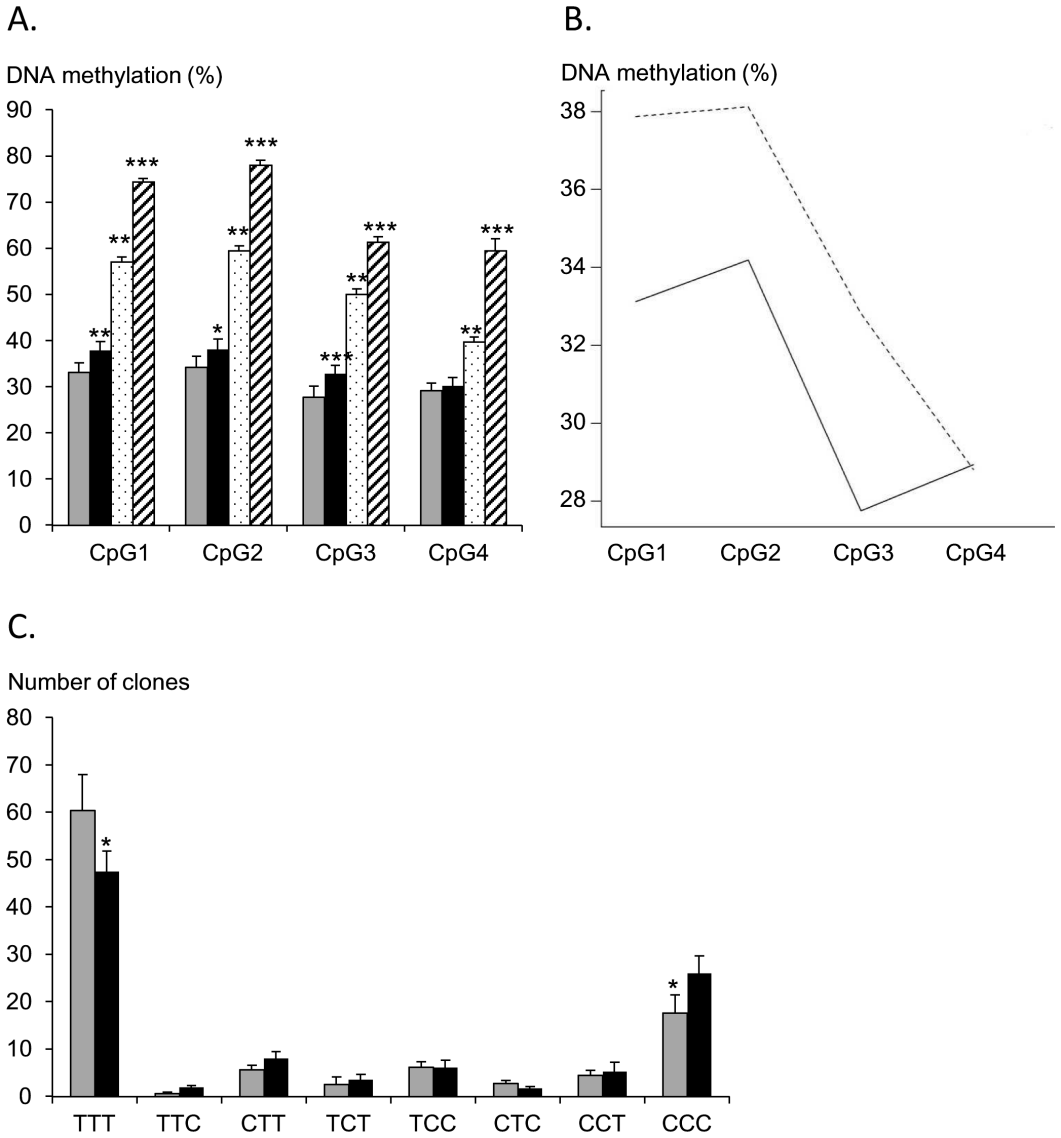
Average methylation levels of the four CpG in mammary or liver DNA. **(A)** Genomic DNA from each udder half of the eight cows at the end of P2 (grey box for the right udder half milked twice daily and black box for the left udder half milked once daily), from the mammary gland of pregnant cows (dotted box) or from liver samples (striped box), were treated with bisulfite and the amount of methylated vs non methylated CpG at the four CpG sequences present in the distal region studied was evaluated by pyrosequencing. Mean values and sem for the different samples are indicated using boxes and bars, respectively. Significant differences between each sample and the right udder half of cows milked twice daily during lactation are indicated as follows: *, P<0.05; **, P<0.01; ***, P<0.001 (for ODM and TDM, n = 8; for the mammary gland during pregnancy and the liver, n = 4). **(B)** Interaction plot showing the differences in the methylation levels of CpG1, CpG2, CpG3 and CpG4 between ODM (broken line) and TDM (solid line). Differences were analysed using Tukey's HSD test. They were highly significant for CpG1, CpG2 and CpG3 with p-values of 0.002, 0.018 and 0.001, respectively. The difference for CpG4 was not significant. **(C)** Different methylation profiles observed for the CpG1-CpG2-CpG3 region. For each udder half of four representative cows (7131, 8136, 8164, 8506) milked twice daily (grey boxes) or once daily (black boxes) during P2, the average number of clones observed for each profile, from fully non-methylated (TTT) to fully methylated (CCC) profiles, is indicated. Differences between ODM and TDM are indicated (*, P<0.05, Wilcoxon test).

Four CpG dinucleotides are present within the distal upstream regulatory region of the *CSN1S1* gene: CpG1, CpG2, CpG3 and CpG4 (Figure 1B). After bisulfite treatment of the gDNA extracted from each udder half at the end of P2, and pyrosequencing using the primers described in Table 2, all cytosines which were not within a CpG sequence were converted to T thus demonstrating complete bisulfite conversion. By contrast, with both ODM and TDM, only some C within CpG sequences were converted to T. For each CpG sequence, the ratio between the number of C and the number of T, which reflects DNA methylation, ranged from 28% to 38% (Figure 5A). An analysis of variance including the “animal”, “mode of milking” and “regulatory region (CpG1 to CpG4)” factors and their first-order interactions revealed that they all induced significant differences (P<0.01). Tukey's HSD test was performed in order to gain an overall picture of the differences between these factors, which were visualised using an interaction plot (Figure 5B) and can be summarised as follows. The methylation level of the four regions clustered in two groups, [CPG1-CPG2] and [CPG3-CPG4]. The differences within these two groups were not significant, but all differences between the two groups were significant (P<0.01). CpG3 and 4 are the least methylated of the four CpG.

The DNA methylation level varied as a function of milking frequency. After ODM, an increase in methylation was observed in CpG1, CpG2 and CpG3, when compared to TDM (p-values  = 0.002, 0.02, 0.001, respectively), whereas no differences were observed in CpG4 (Figure 5B). However, the increased methylation of CpG1, CpG2, CpG3 after ODM was very limited when compared to the difference in methylation profiles observed between lactation and pregnancy (Figure 5A) or between the mammary gland and liver (which does not express CSN genes).

In order to obtain information about the combination, within the same DNA molecule, of CpG1, CpG2 and CpG3 which exhibited different methylation profiles between ODM and TDM, PCR products after bisulfite conversion were cloned and sequenced for the four independent cows which were representative and for which we had obtained *CSN* transcript information by RNA-Seq. ODM or TDM biopsies were analysed from each of the four cows (eight specimens). We sequenced between 85 to 95 clones from each specimen and observed the different expected profiles from fully demethylated (TTT) to fully methylated (CCC) and all intermediate states (TTC, CTT, TCT, TCC, CTC, CCT). The number of clones observed for each profile was evaluated and the average values for the four cows are shown in Figure 5C. The sequence most frequently observed with both TDM and ODM was TTT (60.4% and 47.5% of clones with TDM and ODM, respectively), which means that the three CpG are most often simultaneously demethylated. The second major profile observed with both TDM and ODM was CCC (17.6% and 26.0% of clones with TDM and ODM, respectively) which means that CpG are frequently simultaneously methylated. Other profiles were much less frequent (fewer than eight clones). In half udders milked once daily, the number of TTT clones decreased significantly whereas the number of CCC clones increased significantly, thus confirming the increase in methylation after ODM observed by pyrosequencing.

### In the *CSN1S1* distal upstream region, two potential STAT5 binding sites, one of which overlaps with CpG4, interact with STAT5 *in vitro*

CpG4 is methylated at low levels in the mammary gland during lactation when compared with its methylation state during pregnancy or in the liver (Figure 5A), and this methylation state is not modulated by milking frequency. This CpG is located within a potential STAT5 binding site (D1) and close to a second potential binding site (D2) (Figure 1A). The binding activities of these tandemly arranged potential sites were evaluated using EMSA.

**Figure 6:**
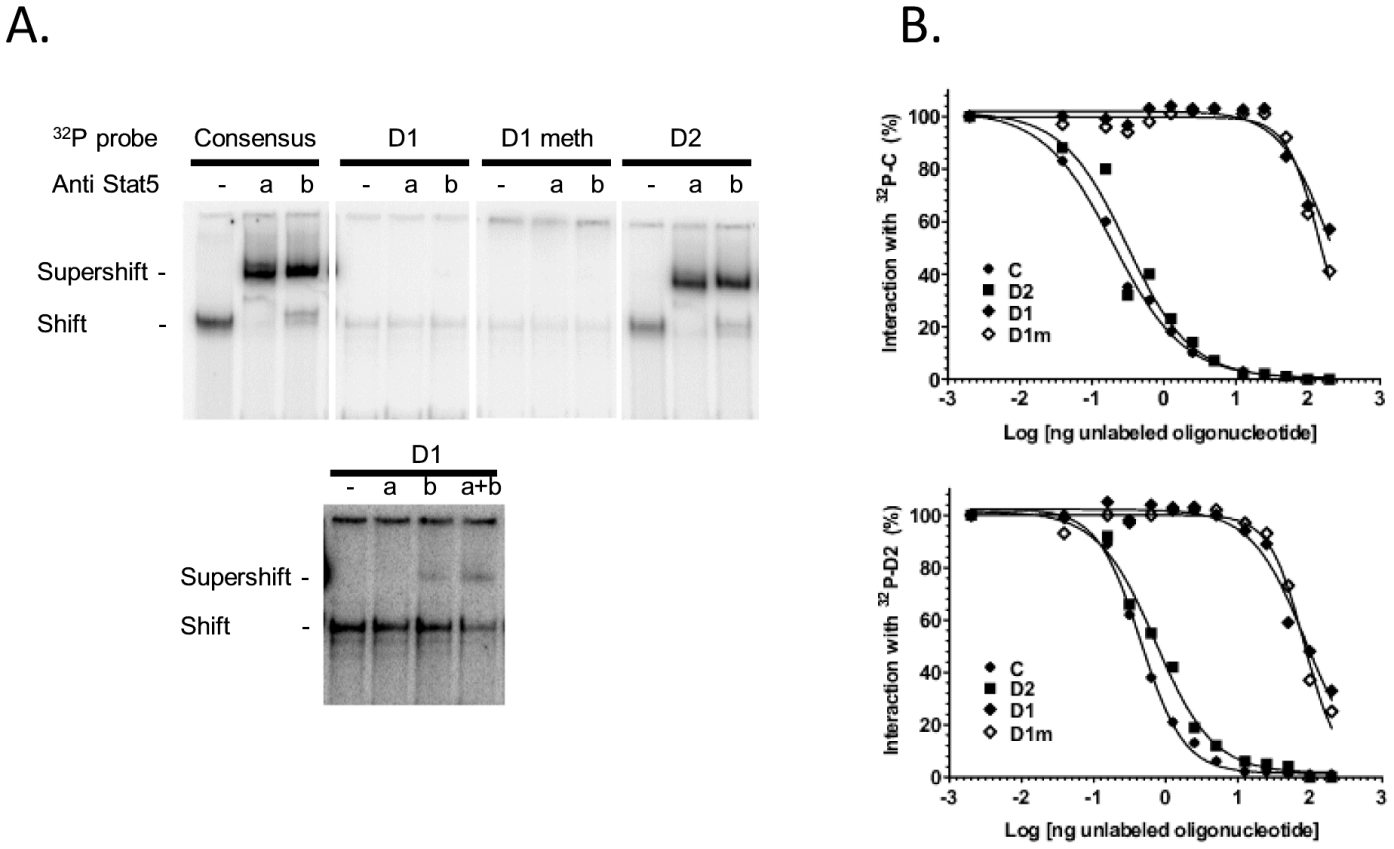
Binding activity of two potential STAT5 binding sites located within the CSN1S1 distal upstream region. **(A)** Representative EMSA. Mammary nuclear extract (22µg) from rabbits in early lactation were incubated with the indicated labelled ^32^P probes in the presence or absence of STAT5a or STAT5b antibodies (1 or 0.5 µg, respectively). Complexes were analysed on non denaturating 5% acrylamide gels in 0.25xTBE. The lower panel is an overexposed image of the upper one to show the weak interaction between D1 and nuclear extracts and the supershifts with STAT5 antibodies. **(B)** Competition assays with increasing amounts of unlabelled C, D2, D1 and D1m using ^32^P-C or ^32^P-D2, are indicated in each graph. Results are expressed as a percentage of the radioactivity bound in the absence of unlabelled oligonucleotides and plotted as a function of the log (ng oligonucleotide) in the reaction.

Mammary gland nuclear extracts incubated with the ^32^P labelled double strand D2 probe (^32^P-D2, corresponding to the more distal potential STAT5 sequence), formed a complex that migrated at a position similar to that observed for the Consensus STAT5 probe, indicated as C in Figure 1B (Figure 6A). The intensity of the signals detected for both complexes was similar. Over 90% of both D2 and C complexes were supershifted with STAT5a and STAT5b antibodies, clearly indicating that these potential STAT5 binding sites interact with both the STAT5a and STAT5b present in the extract. Weaker signals of the same mobility were also observed with ^32^P-D1. They were partially supershifted (15%) by STAT5 antibodies (Figure 6A, lower panel). The signals for ^32^P-D1^m^ were even weaker (Figure 6A). Increasing the amounts of nuclear extracts did not increase the intensity of signals observed with D1 or D1^m^ complexes, thus demonstrating that the weak band shifts were observed under saturating conditions (data not shown). These weak band shift signals could be anticipated by reported analysis of STAT5-DNA binding specificity [35]. The methylation of the CpG within the D1 sequence at most only marginally interferes with this weak *in vitro* interaction.

The specificity and relative binding activities of probes for mammary nuclear extracts were further analysed by competition assays. The binding of nuclear extracts to ^32^P C or ^32^P D2 labelled probes was impaired in the presence of increasing amounts of C, D2, D1, D1 ^m^ unlabelled double strand oligonucleotides (Figure 6B, upper and lower panels, respectively) but not in the presence of irrelevant unlabelled double strand oligonucleotides (data not shown). The amounts of unlabelled C and D2 required to observe half-maximal inhibition (IC_50_) with ^32^P C (Figure 6B upper panel) or ^32^P D2 (Figure 6B lower panel) ranged from 0.2 to 0.7 ng (1.6 to 5.9 nM, respectively). Unlabelled C or D2 therefore competed with their homologous labelled probes with similarly high binding activities, in the nanomolar range. The IC_50_ values for D1 and D1^m^ were similar (within the 95% confidence limit). They were 2-log higher than those of unlabelled C or D2 when the probe was ^32^P D2, and 3-log higher when the probe was ^32^P C. These results were in line with the low binding signal of ^32^P-D1 to the extracts, when compared to that of ^32^P-C or ^32^P-D2 (Figure 6A).

The binding potency of D1 in homologous competition with ^32^P-D1 differed by less than one log from that of D1m (data not shown). The methylation of each strand alone, or both strands together, produced similar profiles, further indicating that methylation only marginally affected the binding potency of the D1 site when assayed independently (data not shown).

Taken together, these results showed that the distal upstream region of *CSN1S1* exhibits a low affinity STAT5 binding site (D1), independent of its methylation status, and a high affinity STAT5 binding site (D2), which might contribute to regulating gene expression in response to prolactin (PRL) and could play an important role in pregnancy and lactation during which PRL levels in the blood vary.

## Discussion

Unilateral ODM induces a major reduction in milk yield and this could reach 37% under our experimental conditions, as already reported [1], [2], [36], and is not fully restored when TDM is resumed during P3. In our experimental design, paired specimens from the same animal were compared so that subtle differences could be observed (7% decrease in P3). This differed from other experimental designs, which compare the effects of bilateral ODM versus TDM on different animals [20].

The variations in milk yield induced by ODM were similar to those affecting the protein and casein yields, but did not fully parallel those of milk protein contents. They were in line with variations already described [37]. Our data on variations in milk CSN yield and content after ODM are the first data to be obtained in cows and differ from those obtained in goats [36]. The lower milk yield during P3 after ODM (7%) suggests a long term reduction in total protein synthesis by udder halves milked once daily. This hypothesis was validated by a decrease in milk protein mRNA including all *CSN* as well as *α-lactalbumin*, whose gene is located on another chromosome. It is however interesting to note that transcript variations, reaching 50% in some cases, were much more important than protein variations. These data are an extension to those previously obtained that allowed us to describe gene networks which vary with ODM and which used a similar experimental design and microarrays for a limited number of genes [2].

Suprisingly, our data further showed that RNA-Seq reads corresponding to the non CSN genes included in the CSN cluster (*STATH* and *ODAM*) were detected at lower levels in udder halves milked twice daily, when compared to those of the *HSTN* gene. Until now, of these three genes, which are expressed in several secretory tissues of epithelial origin [38], [39], the *STATH* and *ODAM* genes had been shown to be expressed in the mammary glands of mouse and humans [8], [40]. *HSTN* expression had not been detected in human lactating mammary gland [8]. In the bovine, a chimeric *HSTN-STATH* transcript was recently described [41], and from its proximity to the CSN2 gene it was inferred to be expressed in the bovine mammary gland during lactation [42] although it has not yet been described [11]. Our RNA-Seq data did not enable identification of this chimeric transcript but demonstrated for the first time that an *HSTN* transcript was clearly expressed in the bovine mammary gland during lactation. The discrepancy between our results and previous results might be due to the fact that, in the bovine, RNA-Seq data have only been obtained from milk somatic cells [11], and that in primates, breast RNA were not extracted from biopsies but from milk fat [8], [43]. Another reason may be that we did not restrict our analyses to polyA+ RNA but extended them to Ribosomal RNA depleted RNA. Our data further indicate that the variations in RNA-Seq reads, corresponding to the three non *CSN* genes of the *CSN* cluster and induced by ODM as compared to TDM, were not similar. The number of *HSTN* reads was significantly higher and the variation after ODM paralleled that of *CSN*, whereas *ODAM* only tended to change and *STATH* was not modified.

Within the *CSN* gene cluster, several regulatory regions involved in the mammary expression of each *CSN* gene or the salivary gland expression of the *HSTN* gene, have been described [4], [44], [45]. Potential regulatory regions have also been located in intergenic conserved regions [14]. Among all those regions, we can confirm that the distal upstream region of *CSN1S1* is only methylated to 30% in the mammary gland during lactation, when the gland is milked twice daily [4]. This is a low methylation level, when compared with that in the liver which does not express the *CSN1S1* gene, and it is interesting to note that the methylation level of this region increases in a context of ODM and mastitis when *CSN1S1* expression decreased. Similar relationships between the methylation of regulatory regions and gene expression in the mammary gland have previously been described for proximal regions of the *CSN* genes in the mouse mammary gland during lactation [14] and regarding regulatory regions of another milk protein gene, *WAP*, in the rabbit mammary gland during lactation [46]. This *CSN1S1* distal region is therefore likely to play a key role in regulating the *CSN1S1* gene.

It is interesting to note that, on average, 60% of DNA strands exhibited a fully demethylated distal regulatory region in a mammary gland milked twice daily (Figure 5C). In a population of diploid cells, the distribution of these demethylated copies per cell can vary between two extremes. At one extreme, two copies of demethylated DNA may be present simultaneously in 60% of cells, while in the remaining 40% of cells both copies are methylated. In that situation, if methylation of this region is associated with a silencing of the *CSN* gene, as suggested by the findings of Vanselow *et al*. [4], only 60% of cells will express the *CSN* genes. At the other extreme, one copy of demethylated DNA is present in every cell and both copies are demethylated in 20% of cells. This latter hypothesis, under which all cells are expected to express one copy of the *CSN* gene, fits better with our histological findings (Figure 3) and *in situ* hybridization results, which revealed an accumulation of *CSN* transcripts in a large proportion of luminal epithelial cells during lactation [47].

After ODM, the marked fall in *CSN1S1* transcript levels (around 50%) was only related to significant but weak increases in the DNA methylation of CpG1, CpG2 and CpG3 from 33 to 38%, 34 to 38% or 28 to 33%, respectively. While methylation/de-methylation events are likely to be part of this overall control, numerous phenomena also contribute to a reduction in milk synthesis during ODM. ODM has been shown to decrease mammary blood flow, induce capillary derecruitment and decrease nutrient uptake [48], [49]. Moreover a reduction in milk yield may also result from an inhibition mechanism due to the feedback inhibition of lactation and/or a physical effect (increase in intramammary pressure, modifications to tight junction openings) [3]. In most cases, this 4–5% increase in methylation levels targeted the three CpG simultaneously (Figure 5C). Following the same line of argument as above, if methylation targets only one allele per cell, such a 4–5% increase in methylation would be observed at most in 8–10% of cells. Such a hypothesis now needs to be studied following the analysis of methylation at a cellular level.

DNA methylation is a relatively stable event and one might expect that the methylation state after ODM would remain until the next cell division, when the marks of methylation might be passively erased. Mammary cell renewal during lactation is limited [50] and the 4–5% increase in methylation observed at the end of ODM may be therefore linked to the 7% long term decrease in milk yield, which continued after TDM was resumed. This increase in the DNA methylation of CpG1, CpG2 and CpG3 after ODM was only modest when compared to the differences between lactation (TDM) and pregnancy. One can argue that these differences are related to different cell populations as the structure of the mammary tissue changes deeply between pregnancy and lactation with a large increase in the number of epithelial cells within the stromal tissue. However, the stromal tissue only brings a moderate contribution in terms of cell nuclei and one can consider that most of the genomic DNA is brought by the epithelial compartment of the mammary tissue in pregnancy as well as in lactation. However, it is noteworthy that, unlike the differences observed between ODM and TDM, those in methylation profile between pregnancy and lactation concern all four CpG, of which CpG4 is the most demethylated. CpG4 may therefore play a crucial role in controlling *CSN* gene expression during pregnancy and lactation, and because its methylation profile is not altered by ODM, it may contribute to the sustained expression of the *CSN* gene after ODM.

The specific role of CpG4 might be further strengthened by the fact that it is located within a weak STAT5 binding site (D1). Weak Stat5 binding sites have often been shown to play key role in gene regulation [29], [51]–[53] and methylation of CpG within or close to the binding site have been already described [54], [55]. Although only a very limited variation in binding affinity is observed when CpG4 is methylated, this interaction may be driven by the nearby high affinity STAT5 binding site (D2). Indeed, the two tandemly arranged STAT5 binding sites, D1 and D2, which fulfill the nucleotide sequence requirement for tetramer formation [29], [56]–[58] may create a cooperative high affinity binding site [29], [52], [53], [59]. These binding sites could therefore play important roles in modulating gene expression when the activity and contents of STAT5 in nuclear extracts vary between pregnancy and lactation. They could also play a role after unilateral ODM as both STAT5 activity, evaluated in term of the level of STAT5 phosphorylation (STAT5-P), and contents have been reported to decrease [37]. However, in cellular extracts from half udders milked twice daily and sampled at least 12 hours after milking, faint and variable signals were detected (data not shown), when compared to those observed in mice (which nurse their pups all day long). Similar results have been previously described [60]. Such variations might be due to the fact that STAT5 phosphorylation is transient (around one hour after PRL activation triggered by milking) and STAT5-P may already be down-regulated several hours after milking. Our assay on cellular extracts was not quantitative for such weak signals and the small size of specimens did not enable the preparation of nuclear extracts, a STAT5-P immuno precipitation and a comparative study between TDM and ODM (in which similar or lower signals are expected). However, RNA-Seq results did not reveal any differences in *STAT5A* or *STAT5B* transcripts (data not shown), in line with the absence of variations in STAT5 or phosphorylated STAT5 previously observed in goats [61].

These STAT5 binding sites may however be more or less accessible to STAT5, depending on the methylation state of the nearby CpG1 and CpG3. Whereas they may correspond by themselves to opportunistic STAT5 binding that does not yield activation of neighboring genes [62], together with other STAT5 binding sites or even with other responsive elements located closer to the *CSN1S1* transcription start site, they may concur to form complexes containing multimers of STAT5 and to induce chromatin loop rearrangements as it has already been described for the mouse *CSN2* gene [44].

These STAT5 binding sites are also some 100 bp away from the RNA-Seq antisense reads that have been found in the mammary gland during lactation but not during pregnancy or in other reproductive organs such as the ovary (preliminary data). They may therefore contribute to the specific expression of this RNA during lactation, or conversely, this RNA may play a role in the chromatin structure of the region, a hypothesis that deserves further investigation.

## Conclusions

The results described above reveal that an upstream region of the *CSN1S1* gene is differentially methylated as a function of milking frequency in primiparous heifers with an increase in methylation being seen after ODM. They suggest that such epigenetic modification may contribute to the long term differential expression of the *CSN1S1* gene after ODM. Because DNA methylation is mainly passively erased through cell division, this profile is expected to be maintained until intense cell proliferation occurs, later in lactation or even at the next lactation. In multiparous cows, DNA methylation profiles might therefore differ from those observed during the first pregnancy, and more variability between cows might be expected. Further experiments on a larger set of animals are now required to determine whether DNA methylation can be used as a predictive marker of milk production recovery after once daily milking. Our findings also revealed the existence of a new transcribed region upstream from this distal regulatory region.
